# Combining Catalytic Microparticles with Droplets Formed by Phase Coexistence: Adsorption and Activity of Natural Clays at the Aqueous/Aqueous Interface

**DOI:** 10.1038/s41598-017-03033-z

**Published:** 2017-06-12

**Authors:** Fatma Pir Cakmak, Christine D. Keating

**Affiliations:** Department of Chemistry, Pennsylvania State University, University Park, Pennsylvania, 16802 USA

## Abstract

Natural clay particles have been hypothesized as catalysts on the early Earth, potentially facilitating the formation of early organic (bio) molecules. Association of clay particles with droplets formed by liquid-liquid phase separation could provide a physical mechanism for compartmentalization of inorganic catalysts in primitive protocells. Here we explore the distribution of natural clay mineral particles in poly(ethylene glycol) (PEG)/dextran (Dx) aqueous two-phase systems (ATPS). We compared the three main types of natural clay: kaolinite, montmorillonite and illite, all of which are aluminosilicates of similar composition and surface charge. The three clay types differ in particle size, crystal structure, and their accumulation at the ATPS interface and ability to stabilize droplets against coalescence. Illite and kaolinite accumulated at the aqueous/aqueous interface, stabilizing droplets against coalescence but not preventing their eventual sedimentation due to the mass of adsorbed particles. The ability of each clay-containing ATPS to catalyze reaction of o-phenylenediamine with peroxide to form 2,3-diaminophenazone was evaluated. We observed modest rate increases for this reaction in the presence of clay-containing ATPS over clay in buffer alone, with illite outperforming the other clays. These findings are encouraging because they support the potential of combining catalytic mineral particles with aqueous microcompartments to form primitive microreactors.

## Introduction

Clay minerals, which are composed of aluminosilicates with layered structures, are major components of soils and sedimentary rocks and among the most abundant minerals at the surface of the Earth^1^. Clays have found a wide variety of applications since ancient times including ceramics^2^, electrochemistry^3^, organoclay/polymer nanocomposites^4^ and as catalysts in chemical reactions^5–7^. The availability of clay minerals and their potential for catalytic activity led to the proposal of these materials as inorganic catalysts for chemical evolution of biomolecules on the early Earth^8^. Since then clay particles have been demonstrated as catalysts for polymerization of activated nucleotides and amino acids^9^, lipid self-organization^10–12^ and many other possible prebiotic reactions^11, 13, 14^.

An important step in the transition from nonliving towards living matter is thought to be compartmentalization of molecules and of reactions^15, 16^. Candidates for early-Earth compartments include crevices or pores in rocks^17^, self-assemblies of lipids or simpler amphiphiles to form vesicles^18–23^, and aqueous droplets formed by liquid-liquid phase separation^15, 21, 24^. Combining catalytic mineral surfaces with compartments such as amphiphile vesicles or droplets formed by aqueous/aqueous phase coexistence is therefore appealing as primitive protocell models. Szostak and coworkers reported that clay minerals accelerate the spontaneous conversion of fatty acid micelles into vesicles and some clay might get encapsulated within the vesicles during the process^10, 12^. Because the mechanism(s) of particle encapsulation within droplets of a biphasic system (partitioning and interfacial adsorption) may differ from those for amphiphile vesicles (entrapment during self-assembly), we reasoned that it might be possible to collect particles more efficiently using droplets. Here we explore the possibility of forming clay-containing or clay-coated droplets in aqueous biphasic solutions. Such structures could in principle combine compartmentalization and catalytic properties with relevance to early Earth scenarios and also potential biotechnological applications as microreactors. A possible challenge is that adsorption of polymeric solutes to the mineral surface could inhibit its catalytic activity. The immiscible aqueous phases that result upon demixing in aqueous polymer solutions provide compartments with different chemical and physical properties between which solutes such as biomolecules (or their prebiotic precursors) can partition. Consequently, aqueous two-phase systems (ATPS) have been used for biomolecule separations^25, 26^, food science^27, 28^, and biomimetic compartmentalization^16, 18, 29^, and have been proposed as simple protocell models^15, 24, 30^.

Clay microparticles can adsorb at, and stabilize, oil/water and water/air interfaces to form oil-in-water Pickering emulsions and foams^31–33^. Emulsions based on oil/water have been stabilized by clay particles^34, 35^ or clay particles with surfactants^32^ and chemical modifications^36, 37^. The driving force for particle assembly at these fluid interfaces is thought to be reduction of interfacial tension. The energy difference due to interfacial adsorption (Δ*E*) depends on the interfacial tension (*γ)*, the particle radius (*R*), and the contact angle (*θ*). For spherical particles, the energy to remove a particle from the interface can be written as:

1 $${\rm{\Delta }}{E}_{{\rm{sphere}}}=-\pi {R}^{2}\gamma {(1-|\cos \theta |)}^{2}$$

Emulsion stabilization is generally observed when particles adsorb strongly to the interface and are preferentially wet by one of the phases, which then serves as the continuous phase^38–40^. Unmodified clays are generally hydrophilic, and consequently form oil-in-water emulsions, while hydrophobically-modified clays can stabilize water-in-oil emulsions^31, 36, 39, 41^. The much smaller interfacial tensions and less obvious wettability preferences at aqueous/aqueous as compared to oil/water interfaces makes stabilizing water-in-water emulsions more challenging. Nonetheless water-in-water Pickering emulsions stabilized by latex beads^42, 43^, protein particles^44^, triblock copolymers^45^, and synthetic clay particles^40^ have been produced.

Clay particles are generally nonspherical, consisting of stacked sheets, which is of interest because ΔE depends on particle shape. For thin platelet-shaped particles, the $(1-|\cos \,\theta |)$ term in the ΔE expression is not squared, resulting in stronger interfacial adsorption than for spheres of the same radius, except when θ = 90°^38, 40^. Erne and coworkers recently demonstrated stabilization of water-in-water emulsions formed from Dx 100 kDa and gelatin 100 kDa aqueous biphasic system using a synthetic clay, gibbsite, composed of aluminum hydroxide nanoplates^40^. Despite interfacial tension of just ~4 μN/m at their aqueous/aqueous interface, these authors were able to form stable Dx-in-gelatin and gelatin-in-Dx all-aqueous emulsions using hexagonal nanoplates only ~170 nm wide and ~7 nm thick, underscoring the favorable interfacial adsorption and reduced sensitivity to contact angle of platelet morphologies. Larger gibbsite plates also adsorbed to the Dx/gelatin aqueous/aqueous interface but due to their greater mass caused droplet sedimentation rather than the well-dispersed emulsions observed with smaller particles^40^.

**Figure 1:**
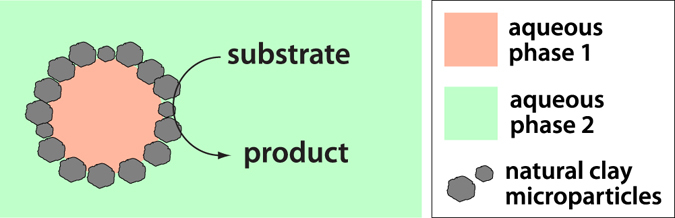
Natural clay particles catalyzing reactions in aqueous two-phase systems.

Here, we examine the behavior of natural clay mineral particles in an ATPS formed from PEG (8 kDa) and Dx (10 kDa) (Fig. 1). A phase diagram for the PEG/Dx system is shown in Supplementary Fig. S1. We selected this system for the chemical simplicity and relatively low molecular weights of the polymers and because the PEG/Dx system has been extensively characterized^15, 24, 26, 46^. Although this specific ATPS is not likely to be early Earth relevant due to high total polymer concentrations, it provides an aqueous/aqueous interface with controllable interfacial tension to evaluate interfacial particle assembly and allows the impact of possible polymer-clay interactions upon particle assembly and catalytic activity to be explored. Three types of common natural clays were compared: kaolinite, montmorillonite and illite, which differ in their particle size, crystal structures and layering as well as their swelling properties. Particle distribution between the phases, polymer interactions with the particle surface, and microstructure of clay-containing biphasic aqueous systems were investigated. Despite the very similar composition and surface charge of these different clays, they behave quite differently in the PEG/Dx ATPS. Clay microparticle stabilized droplets are observed only for kaolinite and illite, both of which show adsorption of the dextran polymer to their surfaces. In contrast, montmorillonite samples did not show significant adsorption of either polymer, and were not able to form particle-coated droplets. Catalytic activities of these clay-containing ATPS were tested in a colorimetric oxidation reaction; reaction rates for clay-containing ATPS samples were similar to polymer-free controls, demonstrating that polymer binding to the clay surface did not prevent catalytic activity. Our findings suggest that natural clay microparticles can be combined with aqueous phase droplets to take advantage of both the compartmentalization capabilities of ATPS and catalytic properties of clays.

## Results and Discussion

### Characteristics of the natural clay minerals

Table 1 summarizes the clays studied in this work, which were selected to include the three main classes of natural clay particles: kaolinite, montmorillonite, and illite^47, 48^. They are all aluminosilicate minerals having layers of tetrahedrally coordinated silicon and octahedrally coordinated aluminum but differ in their number of layers, substitution ion in the layers and interlayer materials^49, 50^. We used two different montmorillonites to investigate the effect of exchange of different cations (Na^+^ and Ca^2+^) in clay minerals. In addition to their primary components these natural clays also contain small amounts of additional minerals as noted in Table 1.

**Table 1:** Charge and size measurements for clay minerals.

Clay Type	Composition^51, 52^	Average Size^a^ (µm)	Polydispersity Index (PDI)^b^	Zeta Potential (ζ) in Buffer^a^ (mV)
Kaolinite (KGa-2)	~96% kaolinite, 3% anatase, 1% crandallite, mica/illite	1.22 ± 0.07	0.32 ± 0.03	−40 ± 1.8
Na-rich Montmorillonite (SWy-2)	~75% montmorillonite, 8% quartz, 16% feldspar 1%, mica/illite	1.37 ± 0.09	0.49 ± 0.07	−45 ± 1.8
Ca-rich Montmorillonite (STx-1b)	~67% montmorillonite, 30% opal-CT, %3 quartz, feldspar + kaolinite	1.61 ± 0.14	0.40 ± 0.13	−34.7 ± 2
Illite (NX-illite)	~60.5% illite, 13.8% illite-smectite mixed layer, 9.8% feldspar, 7.2% kaolinite, 6.6% quartz, 2.1% carbonate	0.84 ± 0.06	0.12 ± 0.13	−31 ± 0.3

^a^Clay particles were suspended in 100 mM HEPES pH 7.51 buffer for measurements.

^b^PDI has applicable range between 0.05 and 0.7, lower numbers indicate higher monodispersity.

Despite the compositional heterogeneity of the mineral samples, all of the clay particles were negatively charged, with zeta potentials in the −30 to −45 range, consistent with their surface chemistries and with previous literature reports^1, 53^. Average particle size for the clays used in this work as determined by dynamic light scattering ranged between 0.48 µm to 1.58 µm (Table 1)^51, 52, 54^. All clay samples were polydisperse in particle size and shape, with values consistent with literature^51, 52, 54, 55^. Field emission scanning electron microscope (FE-SEM) images of representative particles are shown in Supplementary Figs S2 and S3. Hexagonal flake-shaped particles were observed for kaolinite and sheet-like structures for both montmorillonites (Supplementary Figs S2B,C and S3B-C). Illite consisted of small particles as shown in Supplementary Figs S2-D and S3-D.

Bulk partitioning experiments of clay particles in 15%/15% and 20%/20% w/w PEG/Dx were performed by adding clay stock solutions to ATPS with vigorous mixing, after which samples were observed over time (Supporting Figs S4 and S5). Kaolinite- and illite-containing samples showed similar partitioning to the Dx-rich phase, while the montmorillonite samples had cloudy top phases, with Na-rich montmorillonite dispersed throughout the entire sample. Different polymer concentrations only affected the Ca-rich montmorillonite, which partitioned to the PEG-rich top phase in the lower concentration ATPS, and partitioned to the Dx-rich bottom phase in the higher concentration ATPS. Sedimentation of all of the clay particles occurred over time. The observed differences in clay distribution in these bulk ATPS samples suggest that these four types of natural clays interact differently with the polymer-rich aqueous phases, which is somewhat surprising given their similar particle sizes, surface charges and aluminosilicate chemistries.

### Microstructure of clay-containing ATPS

**Figure 2:**
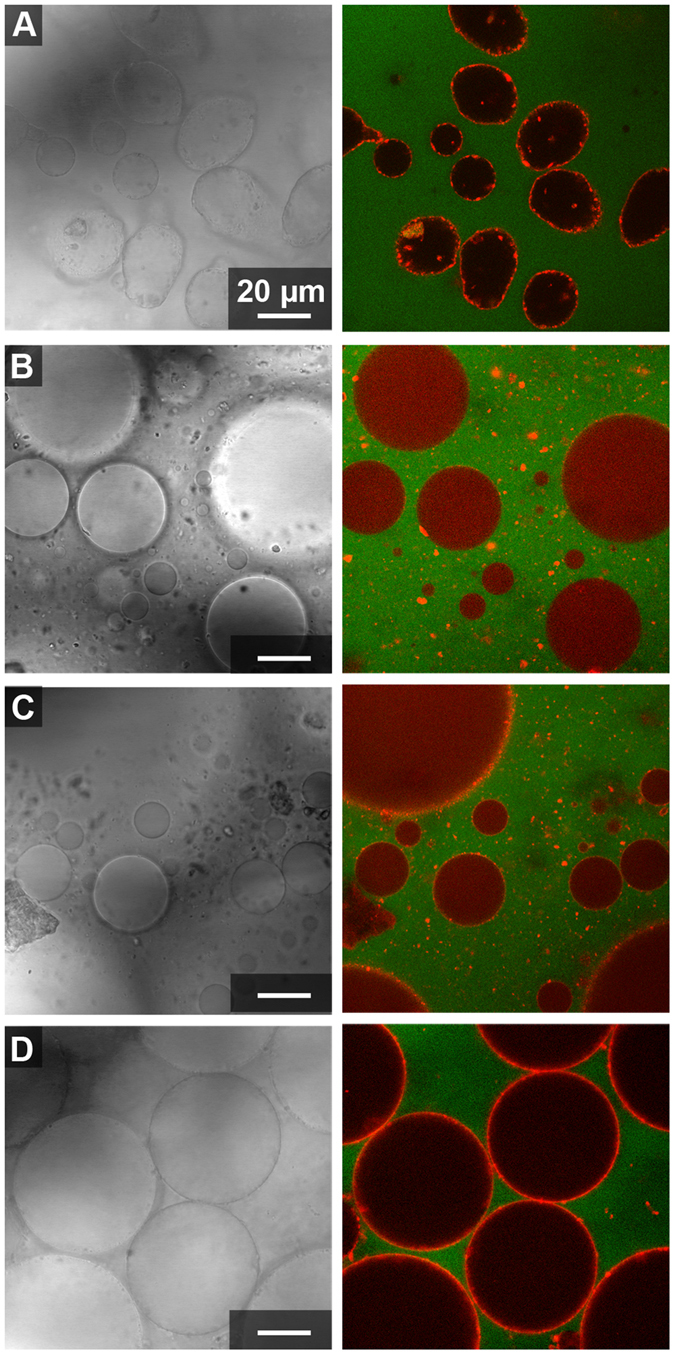
Confocal optical microscope images of clay particles with 1:1 volume ratio from 15%/15% w/w stock PEG-Dx ATPS. Fluorescent-labeled Dx (red) and PEG (green) have been used to image Dx-rich phase droplets and continuous PEG-rich phase. Clay particles appear red in fluorescence images due to adsorption of fluorescently labeled Dx to particle surface. Right images show overlay of fluorescence channels and left images show transmitted light for samples containing: (**A**) Kaolinite, (**B**) Na-rich montmorillonite, (**C**) Ca-rich montmorillonite and (**D**) illite in PEG-Dx ATPS.

**Figure 3:**
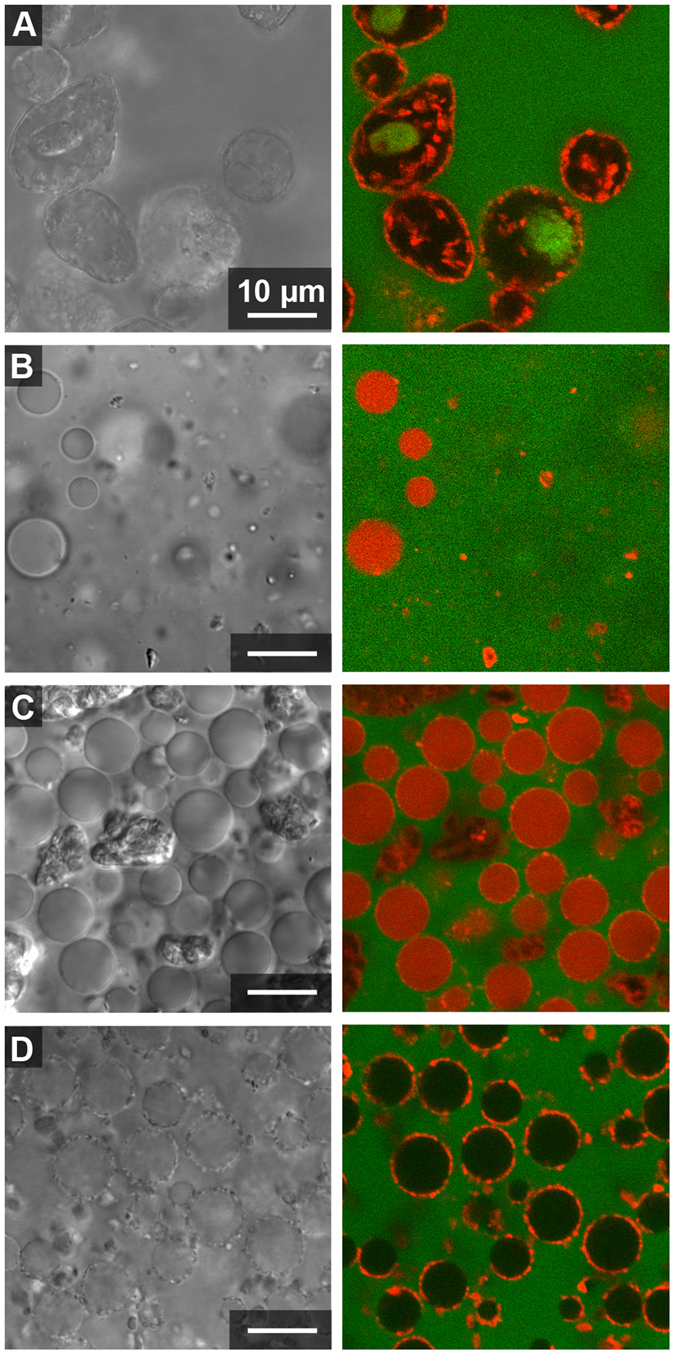
Different volume ratio of ATPS, 20:1 PEG-rich phase: Dx-rich phase from 15%/15% w/w PEG-Dx stock ATPS. Confocal Microscope images of clay particles with fluorescent-labeled Dx (red) and PEG (green) added to aid visualization of Dx-rich phase droplets and continuous PEG-rich phase. Right images show overlay of fluorescence channels and left images show transmitted light for samples containing: (**A**) Kaolinite, (**B**) Na-rich montmorillonite, (**C**) Ca-rich montmorillonite and (**D**) illite in PEG-Dx ATPS.

Clay particles were added to ATPS prepared from stock 15%/15% w/w PEG 8 kDa/Dx 10 kDa by mixing top and bottom phases in desired volume ratios as described in the Methods. The ATPS contained fluorescently-labeled PEG and dextran polymers to help differentiate the two phases in images (the PEG-rich phase was labeled green and the Dx-rich phase was labeled red). ATPS volume ratios 1:1 and 20:1 of PEG-rich to Dx-rich phase volumes were compared. The 1:1 volume ratio was used for simplicity and the 20:1 volume ratio was investigated because most hydrophilic molecules such as proteins, peptides, RNAs or nucleotides partition into the Dx-rich phase and hence for protocells or microreactors applications, a Dx-rich droplet phase would be desirable^18, 56^. Transmitted light and fluorescence micrographs for each of the four clay samples in each of the volume ratios are shown in Figs 2 and 3. An immediately striking feature of these images is the red staining of the clay particles observed most prominently for kaolinite and illite but also to a lesser extent for the two montmorillonites. Kaolinite and illite particles are also found primarily at the aqueous/aqueous interface, while the montmorillonite particles (more visible in the transmitted light images) are found mainly in the PEG-rich phase (Figs 2 and 3).

Literature reports indicate that adsorption of PEG^57, 58^ and Dx^59^ polymers onto clays or other silica and alumina surfaces is likely. In Figs 2 and 3 we observe association of the Alexa647-labeled dextran, but not the Alexa488-labeled PEG, to the clay particles. Polymer adsorption could impact particle wetting by the two aqueous phases and hence any differences in polymer adsorption between the clay types may explain the observed differences in particle distribution in the ATPS. To better understand the observed staining of clays with Alexa647-Dx, we performed a series of control experiments in which the labeled polymers or labels alone (not attached to a polymer) were incubated with each of the clay samples in water (i.e., in the absence of unlabeled polymers). These data are shown and discussed further in Supporting Information (see Supporting Figs S6–S9). We concluded that kaolinite and illite particles are interacting preferentially with labeled Dx, which makes them appear red in fluorescence micrographs, while the montmorillonites do not interact themselves preferentially with either PEG or Dx polymers but a minority population of impurity particles interacts with labeled dextran in the montmorillonite samples. In Fig. 2C, fluorescently-labeled impurity minerals in the Ca-rich montmorillonite sample are found at the aqueous/aqueous interface, but do not provide stabilization against droplet coalescence (note large size and polydispersity of droplets). At the 20:1 volume ratio, these impurity particles are less prominent at the interfaces (Fig. 3C).

The most notable difference between the volume ratios was that larger Dx-rich droplets were observed when more Dx-rich phase was present (compare Figs 2 and 3). Additionally, more labeled Dx is visible in the Dx-rich droplets for the montmorillonite samples at 20:1 as compared to 1:1 volume ratio; this is because the same amount of labeled Dx was present in both samples, and it is more concentrated for smaller Dx-rich phase volume. This is not observed for kaolinite or illite samples due to the strong adsorption of labeled Dx to the particles in those samples. Dx-rich droplets containing kaolinite have irregular shapes indicating arrested coalescence unlike the other samples we have investigated^60^.

Although samples for a given clay type were similar regardless of volume ratio, differences between the four clays are apparent. Kaolinite and illite, which interact preferentially with the Dx polymer, showed greater interfacial accumulation than either montmorillonite and formed essentially complete coatings around the Dx-rich phase droplets (Table 2). Some interfacially-adsorbed clay particles could also be seen in montmorillonite samples, particularly for the Ca-rich montmorillonite sample, although the majority of montmorillonite particles were found in the PEG-rich phase. The interfacial particles here appear to preferentially be non-montmorillonite impurity particles, as evidenced by their strong labeling with fluorescent dextran (see Supporting Fig. 6 and Supporting Discussion). These observations on clay distribution in the ATPS are generally consistent with the differences in polymer adsorption and bulk partitioning in Table 2 and Supplementary Figs S6–S9. Additionally, kaolinite samples had non-spherical clay-coated droplets that contained some interior clay particles and in some cases also what appear to be inclusions of PEG-rich phase within the Dx-rich droplets (see Fig. 3A). Dx-rich droplets in the other samples were spherical, with uniform interiors. As the interfacial area gets reduced during coalescence, surface coverage of droplets increase leading to jamming at the interface without further shape relaxation leading to non-uniform shapes. The non-spherical droplets indicate the presence of arrested coalescence of kaolinite-coated droplets^60, 61^.

**Table 2:** Summary of clay mineral behavior in 15%/15% w/w PEG/Dx ATPS.

Clay Type	Partitioning in bulk ATPS	Interaction with labeled polymer	Microscale distribution in ATPS	Droplet stabilization?
Kaolinite (KGa-2)	Dx-rich phase	Dx	Primarily at the interface with some in Dx-rich phase	Yes (droplets eventually sediment but do not coalesce)
Na-rich Montmorillonite (SWy-2)	no clear preference	—^a^	Interface and PEG-rich phase	No
Ca-rich Montmorillonite (STx-1b)	PEG-rich phase	—^a^	Interface and PEG-rich phase	No^b^
Illite (NX-illite)	Dx-rich phase	Dx	Interface	Yes (droplets eventually sediment but do not coalesce)

^a^No apparent adsorption of either labeled polymer to majority of particles in these samples. Non-montmorillonite impurity particles in these samples interact with labeled dextran; see text and Supporting Information for discussion.

^b^Ca montmorillonite particles in PEG-rich phase sometimes physically block contact of Dx-rich droplets; they do not accumulate at or stabilize the interface.

**Figure 4:**
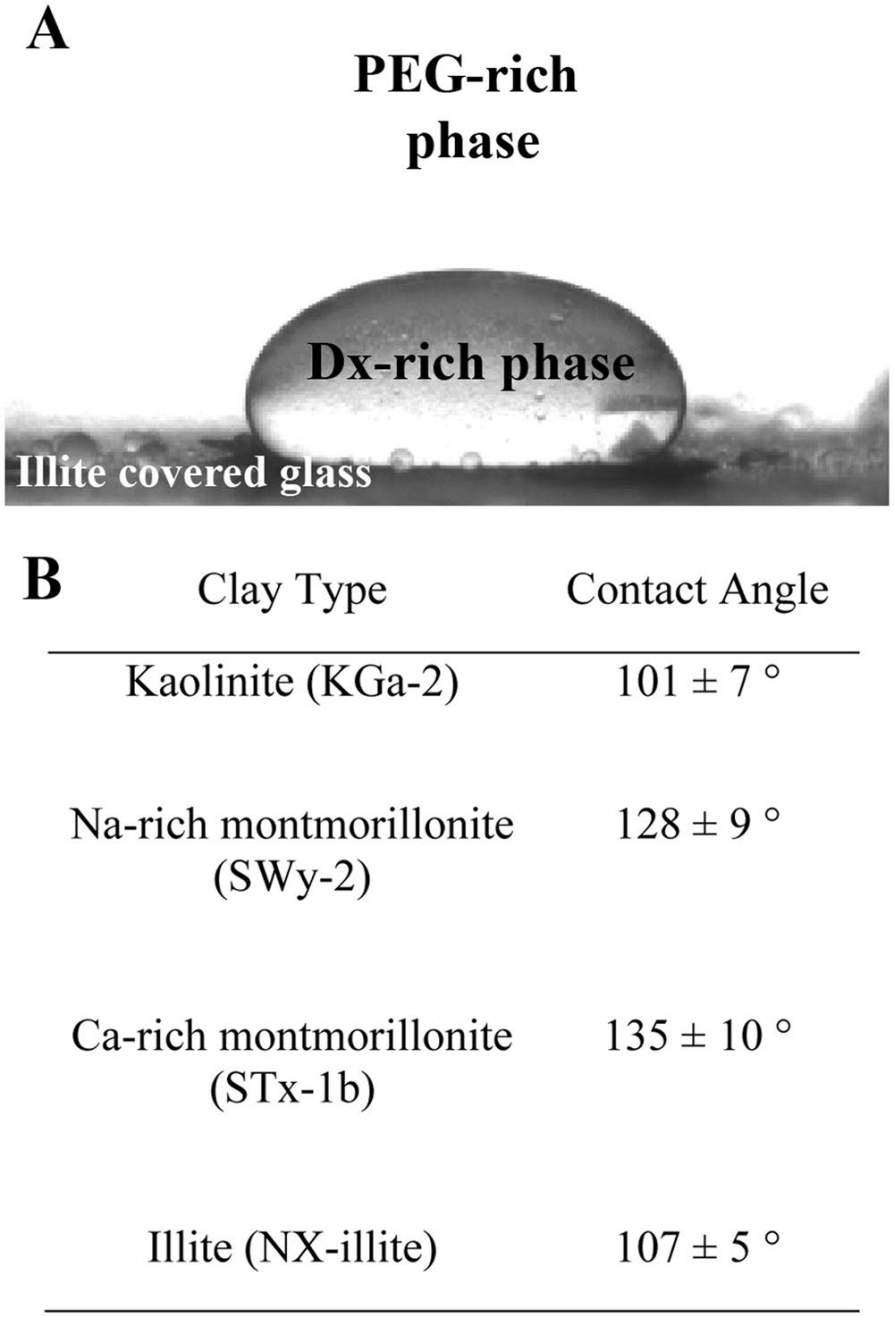
(**A**) Representative photograph of Dx-rich phase droplets sitting on illite-covered glass slides surrounded by a PEG-rich continuous phase, collected to estimate contact angles for clay microparticles in the ATPS, and (**B**) calculated three-phase contact angles from multiple images for different Dx-rich droplets on surfaces that were coated with clay were listed (Error is standard deviation of contact angle measurements).

Over time, illite or kaolinite clay-clad particles sedimented to the bottom of the sample chamber but they did not coalesce even when droplets were in contact. In contrast, droplets in montmorillonite-containing samples coalesced readily upon contact, with stabilization only occurring when accumulations of clay particles in the PEG-rich phase physically prevented Dx-rich droplets from contacting each other (Supplementary Fig. S10). The fact that interfacial coatings of illite or kaolinite can stabilize Dx-rich droplets against coalescence in a continuous PEG-rich phase is perhaps counterintuitive since these clays appear to be wet preferentially by the Dx-rich phase in bulk partitioning experiments, and also are coated with a layer of adsorbed polymer (Table 2). When spherical colloidal particles provide steric stabilization, they are generally preferentially wetted by the continuous phase (Bancroft rule)^39, 62–64^. Indeed, clay particles form oil-in-water Pickering and water-in-oil inverse Pickering emulsions depending on their surface hydrophilicity/hydrophobicity^31, 34, 36^. Our observations are more similar to those of Erne and coworkers for gibbsite nanoplates in a Dx/gelatin ATPS, which stabilized droplets of either phase in the other^40^. Three-phase contact angle estimates from measurements in which Dx-rich droplets surrounded by PEG-rich phase were placed on clay-coated substrates indicated contact angles greater than 90° for all four clay types (Fig. 4 and Supplementary Fig. S11). Kaolinite and illite, which interact with Dx and form clay-clad droplets, had contact angles, *θ* ~ 101 ± 7° and 107 ± 5°, respectively, while estimated contact angles for Na-rich and Ca-rich montmorillonite, which do not show effective droplet stabilization, were 128 ± 9° and 135 ± 10°, respectively (Fig. 4B). We interpret the relative lack of wetting by the Dx-rich phase as a consequence of the similarity between the two aqueous phases and the roughness of the clay-coated surfaces, and in the case of the montmorillonite samples also to the lack of chemical interaction with the Dx polymer.

### Effect of increasing interfacial tension

**Figure 5:**
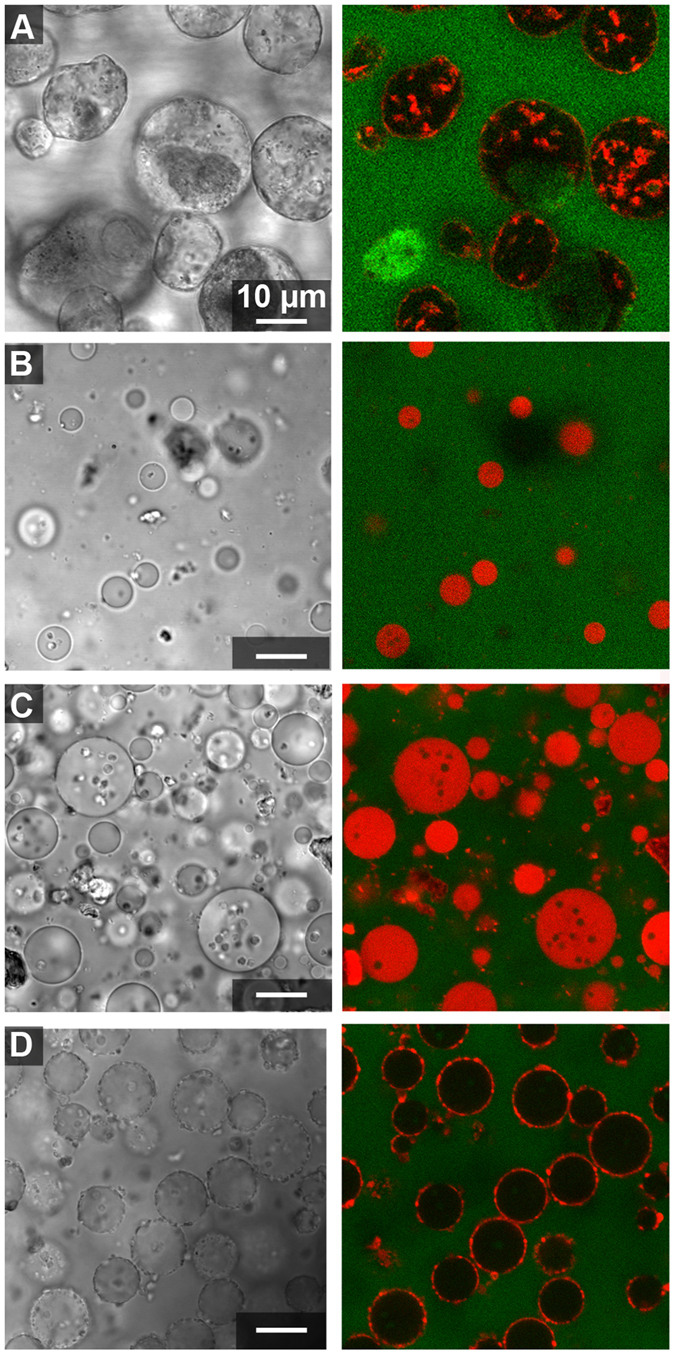
Clay distribution in ATPS with higher polymer concentration. Optical microscope images showing clay particles with 20:1 volume ratio of a stock 20%/20% w/w PEG/Dx ATPS. Left-hand images show transmitted light, and right-hand images show overlay of red and green confocal fluorescence channels for samples containing: (**A**) Kaolinite, (**B**) Na-rich montmorillonite, (**C**) Ca-rich montmorillonite and (**D**) illite. Fluorescent-labeled Dx (red) and PEG (green) have been included to aid visualization of the Dx-rich phase droplets and PEG-rich continuous phase.

We compared clay-containing ATPS prepared using stock solutions containing 15 wt% of each polymer and 20% of each polymer. As expected, the higher polymer concentration led to higher interfacial tension, γ (Table 3). Using spinning drop tensiometer, we found γ = 0.357 ± 0.008 mN/m and 0.746 ± 0.014 mN/m for 15%/15% and 20%/20% w/w PEG 8 kDa-Dx 10 kDa, respectively (see Materials and Methods). We increased the polymer percentage gradually and checked the interfacial results for polymer concentration between 15%/15% and 20%/20% w/w PEG 8 kDa-Dx 10 kDa (Supplementary Fig. S12). Increasing the interfacial tension by a factor of more than two-fold might be expected to increase adsorption of clay microparticles at the ATPS interface. Qualitatively, our results were very similar for the two different polymer percentage ATPS, however (compare Fig. 5, with higher γ, to Fig. 3). Notably, the montmorillonite particles still fail to accumulate appreciably at the interface even at the higher γ. There appears to be more internal structure in the Dx-rich droplets, particularly for Ca-rich montmorillonite, at the higher polymer concentrations. This could be a result of greater solution viscosity in the more concentrated ATPS.

**Table 3:** Physical properties of 15%/15% and 20%/20% w/w PEG/Dx ATPS.

	γ (mN/m)	Sample	wt% Dx	wt% PEG
15%	0.357 ± 0.008	PEG-rich	2.85 ± 0.8	23.46 ± 0.45
		Dx-rich	35.92 ± 0.88	0.36 ± 0.49
20%	0.746 ± 0.014	PEG-rich	2.41 ± 0.82	33.78 ± 0.47
		Dx-rich	40.73 ± 0.86	8.97 ± 0.47

### Inverted emulsions

**Figure 6:**
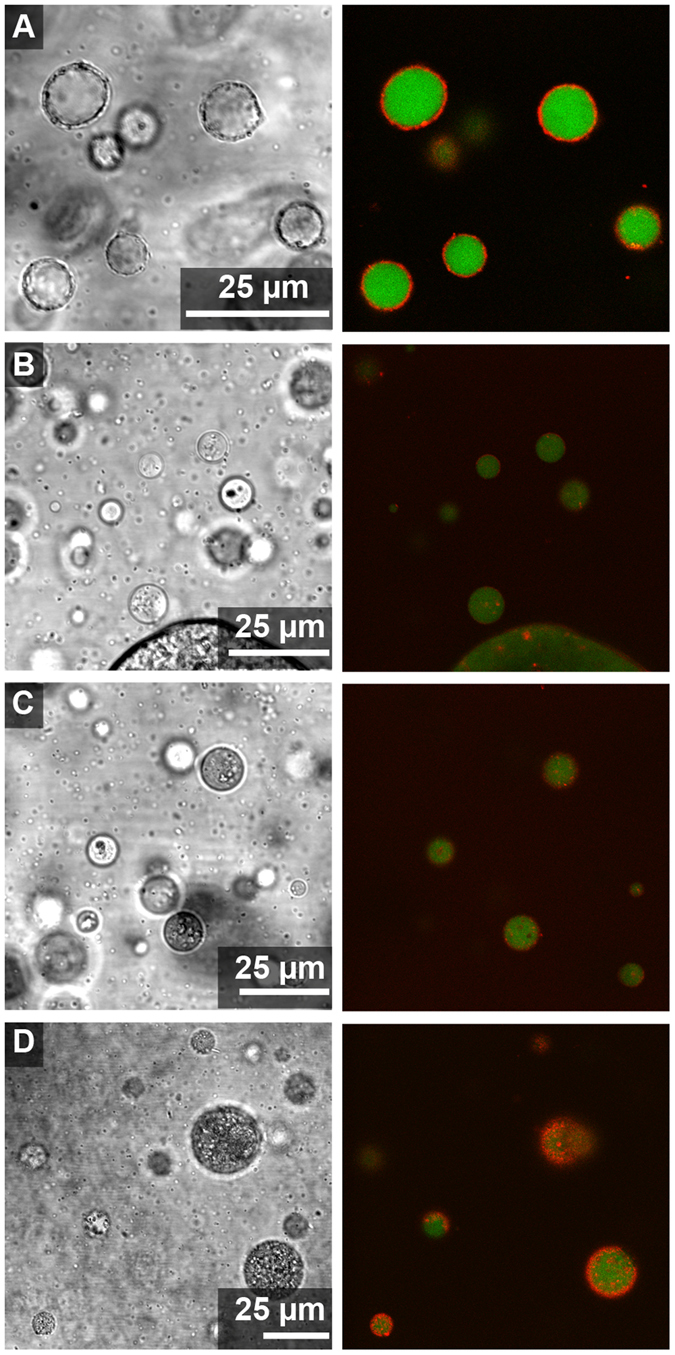
Clay distribution in ATPS having reverse volume ratio. For 20%/20% w/w PEG 8 kDa-Dx 10 kDa using volume ratio of 1:20 PEG-rich phase: Dx-rich phase with (**A**) Kaolinite (KGa-2), (**B**) Na-rich montmorillonite (SWy-2), (**C**) Ca-rich montmorillonite (STx-1b) and (**D**) NX-illite. Labeled Dx (red, 2 μM final concentration) and labeled PEG (green, 2 μM final concentration) have been used for fluorescence imaging. Left-hand images show transmitted light, and right-hand images show overlay of red and green confocal fluorescence channels. Continuous phase is Dx-rich phase while droplets are PEG-rich phase.

Reversing the volume ratio from 20:1 to 1:20 PEG-rich phase:Dx-rich phase led to formation of clay-stabilized PEG-rich droplets surrounded by Dx-rich continuous phase (Fig. 6). Samples containing kaolinite showed relatively spherical, uniform clay-stabilized droplets that resisted coalescence. Na-rich montmorillonite and Ca-rich Montmorillonite did not form stable droplets for the 1: 20 PEG-rich phase: Dx-rich phase ratio (droplets seen in Fig. 6B,C were not stable against coalescence). Samples containing illite formed clay-coated PEG-rich droplets that were more heterogeneous in their coatings and less stable against droplet coalescence than for the 20:1 PEG-rich phase: Dx-rich phase ratio (Fig. 6D). We observed similar trends for both of the ATPS compositions (15%/15% and 20%/20% w/w 10 PEG-Dx ATPS, see Supplementary Fig. S13 for 15%/15%).

### Darkfield imaging to directly visualize clay particles

**Figure 7:**
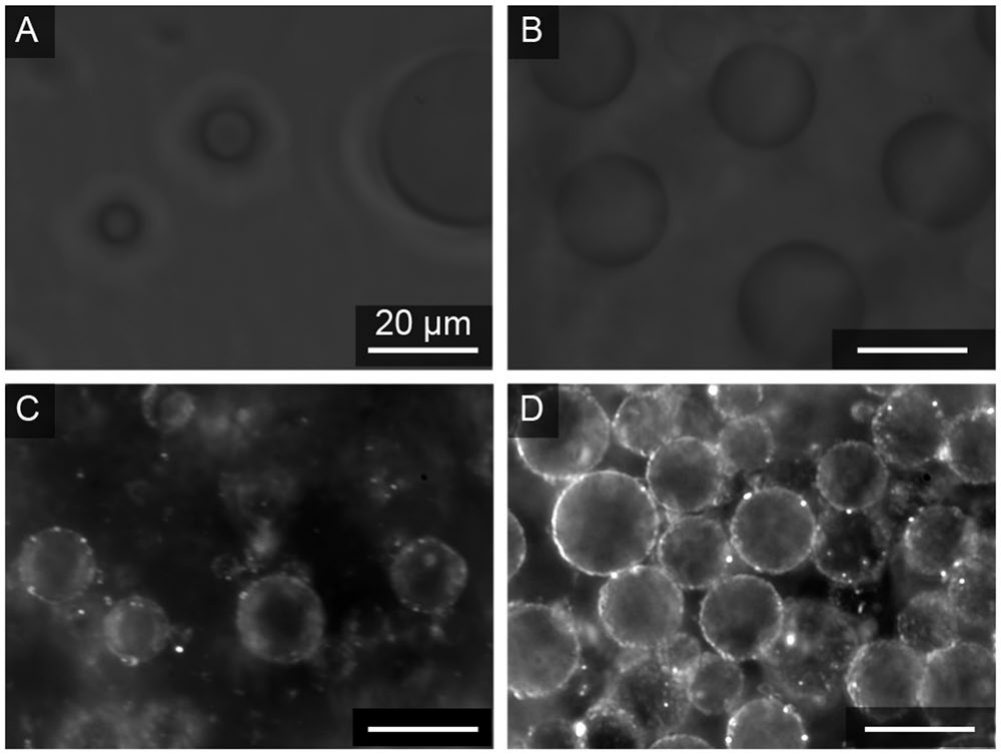
Darkfield optical microscope images showing ATPS ﻿before (**A**,**B**) and after (**C**,**D**) addition of clay microparticles. Kaolinite-stabilized PEG-rich droplets in Dx-rich continuous phase (**C**) (phase volume ratio 1:20 PEG-rich: Dx-rich), illite-stabilized Dx-rich droplets in PEG-rich continuous phase (**D**) (phase volume ratio 20:1 PEG-rich: Dx-rich) are formed. All scale bars represent 20 µm. ﻿Stock ATPS for both samples was 15%/15% w/w PEG 8 kDa-Dx 10 kDa.

Darkfield microscopy was also used to more clearly image clay microparticles at the aqueous-aqueous interface without need of fluorescent labels. Samples without clay have poor contrast at the ATPS phase boundary (Fig. 7A,B), but when clay is present it is apparent at this interface and provides stabilization against droplet coalescence. Figure 7C,D shows PEG-in Dx droplets stabilized by kaolinite and Dx-in-PEG droplets stabilized by illite, respectively. Darkfield imaging for these clay-clad droplet samples is consistent with the fluorescence data shown above, indicating that the fluorescent labels are not playing an important role in particle adsorption at the interface.

### Clay particles as catalysts for a simple reaction

We next evaluated the catalytic performance of the clay microparticle-containing ATPS. Concentration of reactants by surface adsorption has been suggested as one mechanism for surface-catalyzed polymerization^65^. In an encouraging recent report, Mann and coworkers explored the catalytic activity of titania nanosheets sequestered into coacervates used as micro-droplet reactors for photocatalytic degradation^66^. Differences in sequestration of organic dyes into the droplets provided reaction selectivity in that system. There have been no studies as yet of natural clays and their catalytic activity in neutral polymer ATPS like the PEG/Dx system used here. Since Dx was observed adsorbing to the surfaces of the clay particles, competition for adsorption sites could be expected to reduce the effectiveness of clay mineral surfaces as catalysts in ATPS. We nonetheless experimentally tested the feasibility of implementing these systems as catalytically-enabled aqueous phase droplet microreactors. As proof-of-principle, we chose the reaction of *o*-phenylenediamine (OPD) with hydrogen peroxide to form 2, 3 diaminophenazine. This reaction, commonly catalyzed by the enzyme horseradish peroxidase, can be followed spectrophotometrically by product absorbance at 417 nm. Although many colorimetric reagents exist for assaying peroxidase activity, we chose OPD due to its relative hydrophilicity as compared with substrates such as Amplex red or 3,3′,5,5′-tetramethylbenzidine (TMB). Aumiller *et al*. recently reported significant effects of macromolecular crowding agents including PEG and Dx^67^. Substrate hydrophobicity was linked to greater interactions with PEG present in the solution as a macromolecular crowder, and these interactions greatly reduced enzymatic activity due to sequestration of the substrate. These effects were found to be stronger for TMB than OPD in that work.

**Figure 8:**
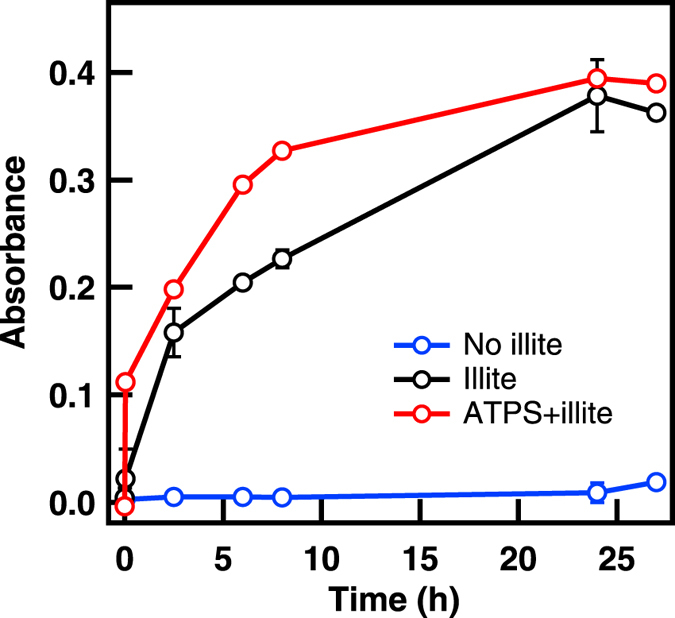
OPD reaction progress. Reaction product absorbance at 417 nm in buffer with substrates without presence of illite (blue), in buffer with illite and substrates (black) and in 20:1 ratio of Dx-rich phase/PEG-rich phase with substrates (red). Hydrogen peroxide was held in excess at 8.8 mM and OPD was 500 µM in 100 mM HEPES buffer, pH 7.5. Error bars represent the standard deviation of the measurements.

In our experiments clay microparticles were used as catalysts for OPD reaction with peroxide in the PEG/Dx ATPS. Reactions were performed in quiescent solutions of 20:1 ratio of Dx-rich phase/PEG-rich phase and followed over time for approximately one day; at each time point samples were centrifuged to sediment clay microparticles before spectrophotometric product determination in the supernatant (Supplementary Figs S14–18). Controls were also performed in buffer and single phases, and in the absence of clays or OPD (Supplementary Figs S14–S16). Quantification of product absorbance at 417 nm indicated that all four clays (kaolinite, both montmorillonites, and illite) had slightly higher activity in the ATPS than in buffer alone (Fig. 8 and Supplementary Figs S17–S18). Illite was most active for this reaction in our experiments, both in buffer and in the ATPS. Na-rich montmorillonite samples also showed good catalytic activity but the product remained associated with the particles and was not distributed in the solution (Supplementary Fig. S19; we note that it was not possible to distinguish between activity and adsorption to montmorillonite vs. its impurity minerals in these experiments). When clay was not present, product was not observed.

The ability of illite-clad ATPS droplets to catalyze this reaction is an encouraging proof of concept for natural clay microparticles providing catalytic capabilities to droplets formed by aqueous-aqueous phase coexistence. Despite adsorption of Dx to the illite surface (see Table 2), reaction rates were slightly higher for illite in the ATPS than in buffer alone. This may be due to the partial Pickering emulsification of the illite/ATPS system, where clay-clad droplets remain suspended for many hours, facilitating reaction of the illite with OPD in solution. We note that the reaction product 2, 3 diaminophenazine did not accumulate in either phase of the ATPS (its partitioning coefficient, K, was 1.01 ± 0.01, where K = C_P_/C_D_ and C_P_ and C_D_ represent the concentrations of the product in the PEG-rich and Dx-rich phases, respectively). Based on its similar structure and smaller size, the OPD substrate is also not expected to accumulate in either phase. Further rate enhancements should be achievable by taking advantage of substrates and/or products that exhibit strong partitioning between the two aqueous phases to facilitate access to the catalytic clay particles.

## Conclusions

The work presented here shows the rich behavior of natural clays in the PEG/Dx ATPS, with surprisingly different observations for kaolinite, montmorillonite and illite particles despite their similar chemical compositions and surface charge. Although bulk Pickering emulsions were not stable over long times due to sedimentation of clay-coated droplets, natural clay microparticles of all types tested adsorbed at the aqueous/aqueous interface to at least some degree. We observed several basic types of geometries that combined clay microparticles with the droplet phase of the ATPS. Kaolinite and illite particles adsorbed readily at the interface to form clay-clad droplets for ATPS with either Dx-rich or PEG-rich dispersed phases. Montmorillonites showed the least accumulation at the interface; montmorillonite-containing Dx-rich droplets were common despite the majority of montmorillonite particles remaining in the PEG-rich phase. Stabilization of Dx-rich droplets against coalescence was possible when relatively large montmorillonite particles accumulated between the droplets; this stabilization mechanism was most effective at high local particle concentrations. We evaluated the ability of ATPS that contained natural clay microparticles to catalyze a simple reaction and found that illite in particular not only retained its catalytic capability despite Dx adsorption to the particles in the ATPS, but in fact the rate was slightly enhanced for the illite-stabilized droplet samples as compared to illite in buffer alone. This is to the best of our knowledge the first paper to describe distributions of natural clay microparticles in ATPS and demonstrate their retention of activity in clay-stabilized ATPS droplets. As such, our findings support the potential of combining catalytic natural mineral particles with aqueous microcompartments to form primitive microreactors, which could be relevant to abiotic chemistry on the early Earth. Although the specific ATPS composition used here may not be directly relevant to early Earth scenarios, a variety of prebiotic polymers were present^15, 68^, and could have participated in aqueous/aqueous phase separation to generate systems similar to those studied here.

## Materials and Methods

### Chemicals and Materials

Three types of clay were supplied from the Clay Mineral Society Source Clays Repository: kaolinite, Warren County, Georgia, USA (KGa-2); Na-rich montmorillonite, Crook County, Wyoming, USA (SWy-2); and Ca-rich montmorillonite, Gonzales County, Texas, USA (STx-1b). NX-illite was obtained from Arginotec, NX Nanopowder, B + M Notenkämper, Munich, Germany. NX-Illite is composed of illite, illite-smectite mixed layer, feldspar, kaolinite, quartz and carbonate^52^.

PEG 8 kDa, dextran 10 kDa, 4-(2-hydroxyethyl)-1-piperazineethanesulfonic acid (HEPES), HEPES sodium salt, sodium bicarbonate, *o*-phenylenediamine tablets, hydrogen peroxide and 2,3-diaminophenazine were purchased from Sigma-Aldrich (St. Louis, MO). Labeled polymer mPEG-NH_2_ MW 5000 was purchased from Shearwater Polymers. Alexa Fluor 647-Dextran 10 kDa, Alexa Fluor 488 labeling kits and 20 mm diameter by 0.5 mm deep press-to-seal silicone isolators were obtained from Life Technologies (Carlsbad, CA). Water was deionized to a resistance of 18.2 MΩ with Barnstead NANOpure Diamond water purification system (Van Nuys, CA) and used for all experiments.

### Preparation of ATPS

Two different stock ATPS were prepared at 10 g total mass: (1) 15%/15% w/w by dissolving 1.50 g PEG 8 kDa, 1.50 g dextran (Dx)10 kDa in 7.00 g 100 mM pH 7.5 HEPES buffer for; and (2) 20%/20% w/w by dissolving 2.00 g PEG 8 kDa, 2.00 g Dx 10 kDa in 6.00 g 100 mM pH 7.5 HEPES buffer. These mixtures were stirred in a glass container at 37 °C for several hours until the polymers were completely dissolved, then either left overnight or centrifuged at 3000 × *g* for phase separation. Different volume ratios of PEG-rich to Dx-rich phases used in the experiments were obtained from phase separated stock solutions and remixed in desired ratios as previously described^24^.

### Phase composition determination

Polymer concentration of PEG-rich phase and Dx-rich phase were determined using refractometry and polarimetry^29^. Abbe Autorefractometer (Leica Geosystems, Norcross, GA) was used to measure refractive index of each phases. Polarimetry measurements were carried out with model No. 343 Polarimeter (PerkinElmer, Billerica, MA). First, concentration of optically active Dx 10 kDa was determined by polarimetry by generating standard curve. Later concentration of PEG was determined by refractometry. Standard curves for PEG and Dx were prepared with known concentrations. Each sample was measured in triplicate using polarimetry and refractometry. Dx contribution to total refractive index was subtracted and remaining refractive index was used to calculate the concentration of PEG in each phases.

### PEG Labeling

mPEG-NH_2_ MW 5000 was labeled with Alexa Fluor-488 in the presence of 0.1 M sodium bicarbonate. The reaction was covered by aluminum foil and stirred at room temperature for 3 h. Free dye was removed using an Amicon 300 MWCO filter with centrifugation at 14000 *g*.

### Dynamic Light Scattering and Zeta Measurements

Suspended stock solution of clay 10 µL was diluted by adding 1 mL DI water for dynamic light scattering measurements except Na-rich montmorillonite, 15 µL of suspended stock was diluted for it. From suspended stock solution of clay 10 µL was diluted by adding 1 mL of 100 mM pH 7.5 HEPES buffer, except Na-rich montmorillonite, 15 µL of stock was diluted with 100 mM pH 7.5 HEPES buffer, for Zeta Potential measurements. Measurements were done in triplicate using a Malvern Zetasizer NanoZS.

### Scanning electron microscopy (SEM)

Clays were imaged by FEI NanoSEM 630 FESEM at 1 keV and 700 eV. In order to prevent charging during imaging the clay particles, which are nonconductive, we followed the method of Veghte *et al*.^51^. Clay particles were impacted by a cascade impactor (PIXE International Corp., Tallahassee FL) over the silicon wafer chips (Virginia Semiconductor Inc., Fredericksburg VA). Then, sparse particles over the silicon wafer were imaged.

### Confocal Fluorescence Microscopy

Images were collected with a Leica TCS SP5 inverted confocal microscope at 63x magnification to image the behavior of clay particles in ATPS. Alexa 488-labeled PEG 5k and Alexa Fluor 647-Dx 10 kDa were excited with a 488 nm argon ion laser and 633 nm, respectively. Samples were prepared by using 20:1 PEG-rich phase: Dx-rich phase and 1:20 PEG-rich ratios. Samples were vortexed before imaging. Glass coverslips used in microscopy were silanized in gas phase with dichloromethylsilane and used with press-to-seal spacer^69^.

### Darkfield Microscopy

Images were obtained with Nikon TE200 inverted microscope using 100x oil objective with iris NA 0.5–1.3 and darkfield oil condenser NA 1.2–1.43 with illumination from a halogen lamp.

### Determination of ATPS interfacial tension by Spinning Drop Tensiometry

Different percentages of ATPS were prepared and phase separated by centrifugation at 5000 × *g* for 10 minutes. Krüss SITE 100 spinning drop tensiometer was used. Heavy phase, Dx-rich phase, was loaded to capillary tube and then light phase (4 µL) was injected. Triplicate tensiometry measurements were taken at 9, 10, 11 and 12k rpm and were in agreement.

### Contact Angle/Pendant Drop

Due to the small size and to the heterogeneity in both particle size and particle shape for each clay sample, it was not possible to determine their contact angles with the liquid phases while they are adsorbed at the liquid/liquid interface. Therefore we followed the method of Vis *et al*.^40^. Clay particles were deposited on glass slides by vertical deposition method^70^. Three-phase macroscopic contact angle between Dx-rich droplets in PEG-rich phase were imaged at least three times per sample, then the contact angle was measured from both left and right sides of the droplets with ImageJ^71^. Each measurement repeated at least three times.

Glass slides (24 mm × 30 mm) were cut in half using a diamond pen in order to fit them into vials. Then they were immersed in 1:1 hydrochloric acid and methanol mixture for an hour. Glass slides were rinsed well and dried with nitrogen. Each slide was placed in a vial with an angle about 30° and dilute solution of clay solution used to fill the vials. They were left on a heating plate at 100 °C overnight to form a layer of clay on the glass slides. Only surfaces well-covered with clay over their entire surface were used. PEG/Dx ATPS (15%/15% w/w) solutions were prepared and allowed to equilibrate as described and then the phases were separated. PEG-rich phase was used to fill the rectangular container and 250 µL pipette tip was filled with denser Dx-rich phase. The tip was immersed in PEG-rich phase containing clay covered glass slide. 3 µL droplets were then dispensed over the clay-covered surface for several times with different glass slides and droplets. Images were taken using a Pendat drop instrument (Ramè-hart Model 295**)** and three-phase contact angle were calculated from both left and right sides of droplets.

### Clay Catalyzed Reaction

**Figure 9:**
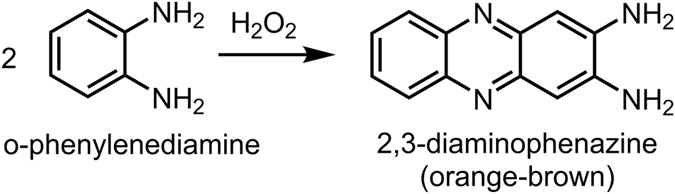
OPD reaction scheme.

Reaction progress was followed using an Agilent 8453 diode array UV-visible spectrometer with Agilent ChemStation software. All measurements were repeated for three times and monitored at 417 nm. Hydrogen peroxide was held in excess at 8.8 mM and OPD was 500 µM in 100 mM HEPES buffer, pH 7.5. OPD tablet was dissolved in HEPEs buffer and used immediately. 10% clay stock was prepared and diluted to 1% in the reaction mixture instead of catalyst. Reaction product (2,3-diaminophenazine) was monitored by UV-Vis at 417 nm (see Fig. 9). As the reaction progressed, the solution changed color to orange–brown^67^.

### Partitioning of Reaction Product

Partitioning of reaction product was determined by measuring the absorbance in each phase. The reaction product, 2,3-diaminophenazine, was dissolved in 1 mL ATPS at a 1:1 volume ratio of PEG-rich:Dx-rich phase with final concentration of at 500 µM. ATPS stock solution was prepared with composition 15%/15% w/w PEG 8 kDa/Dx 10 kDa. After mixing, phases were separated by centrifugation. The concentration of product in each phase was calculated by measuring absorbance of three samples for each phase using an Agilent 8453 diode array UV-visible spectrometer with Agilent ChemStation software. Partitioning coefficients are reported as K = C_p_/C_D_, where C_P_ and C_D_ represent the concentration of product in PEG-rich phase and Dx-rich phase, respectively. Absorbance values were corrected for each phase.

## Electronic supplementary material

Movie 1

Supplementary  Information
