# Supplementary material for: Combining Catalytic Microparticles with Droplets Formed by Phase Coexistence: Adsorption and Activity of Natural Clays at the Aqueous/Aqueous Interface
Source: Sci Rep. 2017 Jun 12;7:3215. doi: 10.1038/s41598-017-03033-z (PMC5468296; doi:10.1038/s41598-017-03033-z)
Supplement: Supplementary file 2 — Supplementary Information [file 41598_2017_3033_MOESM2_ESM.pdf]

Supporting Information

Combining Catalytic Microparticles with Droplets Formed by Phase Coexistence: Adsorption and Activity of Natural Clays at the Aqueous/Aqueous Interface

Fatma Pir-Cakmak and Christine D. Keating*

Department of Chemistry, Pennsylvania State University, University Park, Pennsylvania 16802, USA

Preparation of Clay Stocks: 10 mg of clay was added to 100 µL 100 mM pH 7.5 HEPES buffer to prepare stock solutions except Na-rich montmorillonite, which is added to 150 µL 100 mM pH 7.5 HEPES buffer to suspend it completely. Stock solutions were vortexed until they were suspended.

Bulk Clay Partitioning. For each clay type, 100 mg solid was measured and then added into 1 mL of HEPES pH 7.5 buffer. Then suspended stock solution was added into 10 g of 15%/15% w/w PEG 8 kDa /Dx 10 kDa ATPS. Vials were shaken vigorously and photographs were taken at desired time points.

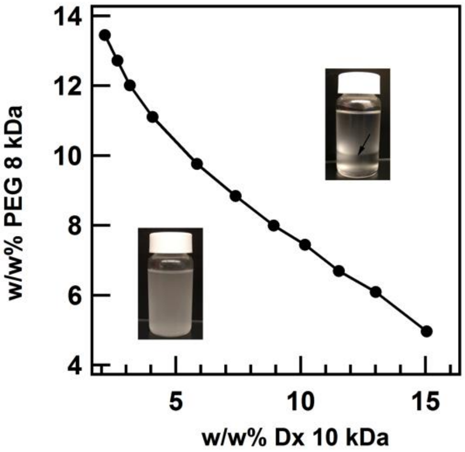

Figure S1. Binodal curve of PEG 8kDa/Dx 10 kDa ATPS. Points were determined by cloud point titration. Below the line, solutions of these polymers exist as a single phase and above the line phase separation occurs.

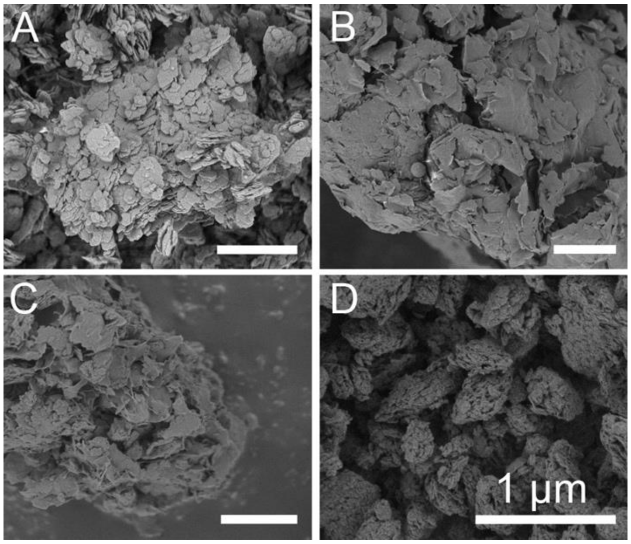

Figure S2. Representative scanning electron microscopy (SEM) images of bulk (**A**) Kaolinite (KGa-2), (**B**) Na-rich montmorillonite (SWy-2), (**C**) Ca-rich montmorillonite (STx-1b) and (**D**) NX-illite.

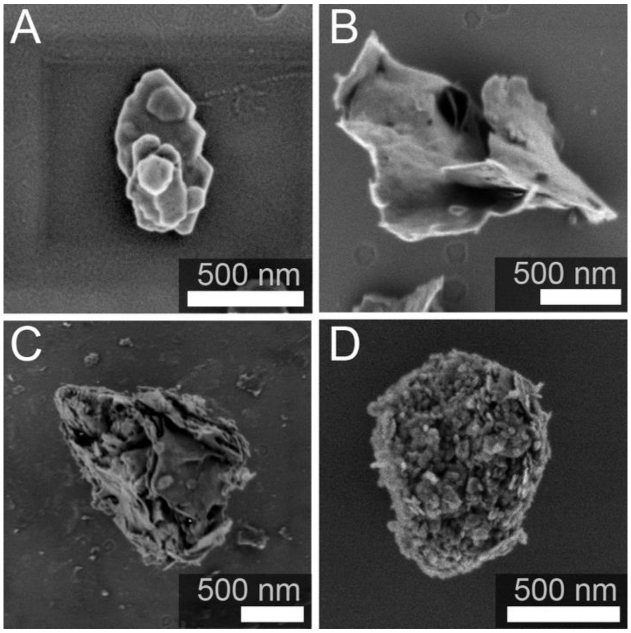

Figure S3. Representative scanning electron microscopy (SEM) images of (**A**) Kaolinite (KGa-2), (**B**) Na-rich montmorillonite (SWy-2), (**C**) Ca-rich montmorillonite (STx-1b) and (**D**) NX-illite particles.

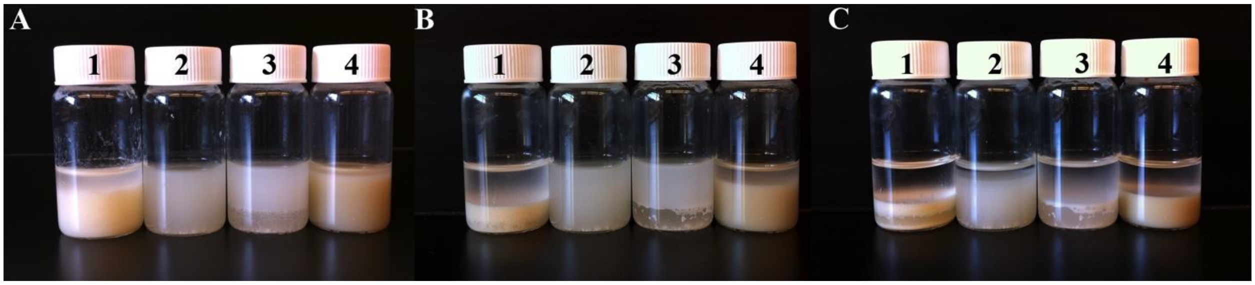

Figure S4. Photographs showing distribution of clay particles in ATPS over time. Time points were taken for each clay type at (**A**) 30 min., (**B**) 3 hours and (**C**) 24 hours. Samples were numbered to indicate which clay is present: (1) Kaolinite, (2) Na-rich montmorillonite, (3) Ca-rich montmorillonite and (4) illite. ATPS composition was 15%/15% w/w PEG 8 kDa-Dx 10 kDa in 100 mM HEPES buffer, pH 7.5, at the native volume ratio of approximately 3:1 PEG-rich (top) to Dx-rich (bottom) phase.

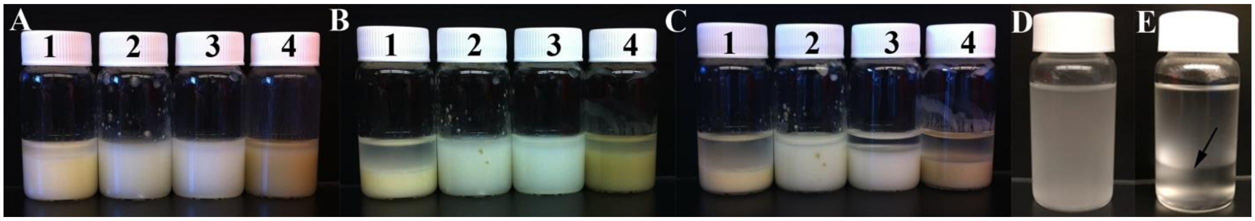

Figure S5. Photographs showing distribution of clay particles in 20%/20% w/w PEG 8 kDa-Dx 10 kDa ATPS over time. Time points were taken for each clay type at (**A**) 30 min., (**B**) 3 h and (**C**) 24 h. Samples were numbered to indicate which clay is present: (1) Kaolinite, (2) Na-rich montmorillonite, (3) Ca-rich montmorillonite and (4) illite. ATPS contained 100 mM HEPES buffer, pH 7.5. Control experiment showing 15%/15% w/w PEG 8 kDa-Dx 10 kDa ATPS without any clay, just after shaking (**D**) and 30 min. later phase separated (**E**).

Supporting Discussion

Interaction of labeled polymers with clay microparticles. Since polymer adsorption can be expected to influence particle distribution in the ATPS, we mixed fluorescently labeled PEG and Dx polymers with each of the clay samples to evaluate their interactions with the clays. By comparing transmitted light images, which show the location of clay particles, and fluorescence emission channels, which show the labeled polymers, we can determine whether either of the dye-labeled polymers is associated with the minerals. In all cases, the background appeared green, indicating free Alexa Fluor 488 (AF 488) labeled PEG in solution (Supporting Fig. S6). Red-stained particles could be seen in all of the samples, indicating the adsorption of Alexa Fluor 647 (AF 647)-labeled Dx to these particles. In the kaolinite sample, all of the particles appear red, while in the other three samples some particles were red while others remained dark, the latter indicating neither labeled polymer has adsorbed appreciably. The greater fluorescence uniformity in the kaolinite sample is consistent with the greater compositional homogeneity of this clay, which contains only 4% of non-kaolinite mineral. In both montmorillonite samples, it appears that the montmorillonite particles themselves do not interact appreciably with either labeled polymer, but a minor component with very regular size and round shape associates with the labeled Dx. We therefore also evaluated binding to likely impurity particles including quartz, feldspar, and orthoclase. These three particle types all interacted preferentially with labeled Dx polymer (Supporting Fig. S7). We concluded that the montmorillonites do not interact themselves preferentially with Dx polymers but rather that the red particles seen in Figure 2 are due to adsorption of labeled Dx to other components (e.g., quartz or other minor components). For the illite sample, the most numerous particles (presumably illite itself, which accounts for 60% of the mineral content) were stained red indicating interaction with the labeled Dx, while a

smaller but still significant fraction of the particles remained unstained and black, the latter showing faint labeling by AF 647-Dx under closer inspection by z-stacking (Supporting Video S1). To rule out the possibility that the observed binding interactions were dominated by the dye moieties rather than the PEG or Dx polymers, we next looked at adsorption of just the dyes themselves in 100 mM HEPES buffer at pH 7.5 buffer (Supporting Fig. S8). No adsorption of either dye was apparent for illite, kaolinite or Na-rich montmorillonite samples. A minority component of the Ca-rich montmorillonite samples showed interaction with AF 647 (note presence of several red-labeled particles in Supporting Fig. S8C, and see also Supporting Fig. S9). We concluded that Ca-rich montmorillonite interactions with fluorescently labeled Dx are the combined result of interactions with the label and the Dx, while kaolinite and illite are interacting primarily with the Dx polymer.

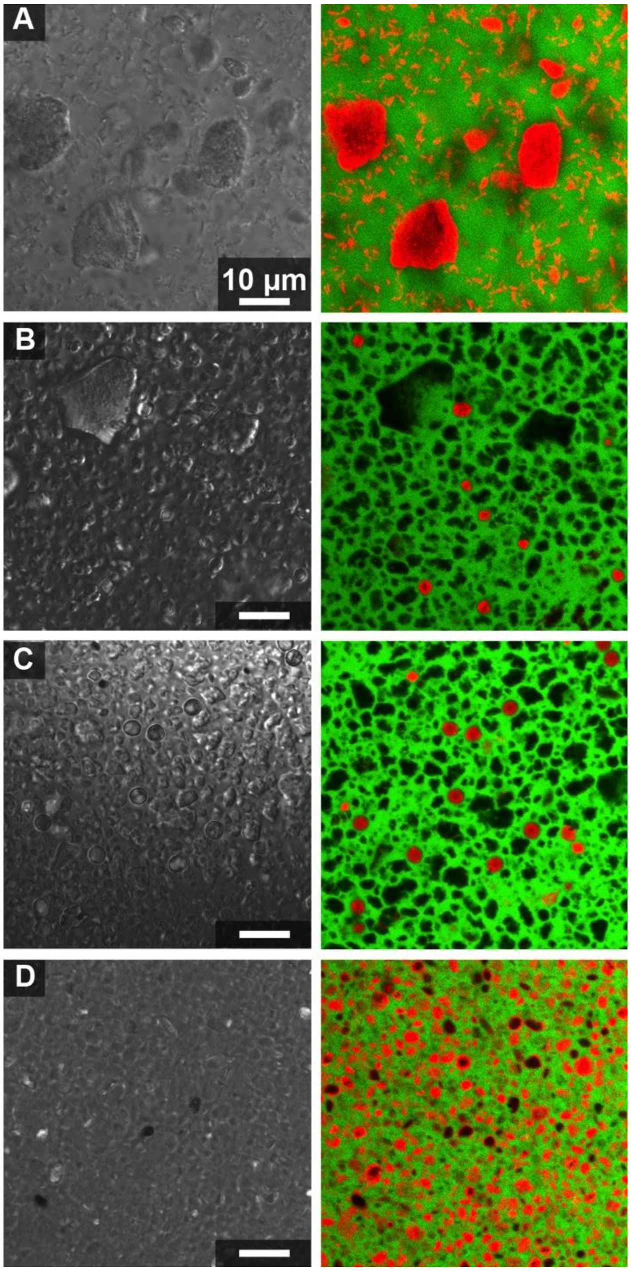

Figure S6. Microscope images showing interaction between clay particles and fluorescently labeled PEG and Dx polymers in water. Images on left and right show location of clay particles (transmitted light) and overlay of red and green confocal fluorescence channels, respectively. Red indicates AF 647-labeled Dx (10 µM), green indicates AF 488-labeled PEG (100 µM).

Samples are: (**A**) kaolinite, (**B**) Na-rich montmorillonite, (**C**) Ca-rich montmorillonite and (**D**) illite. All scale bars are 10 microns.

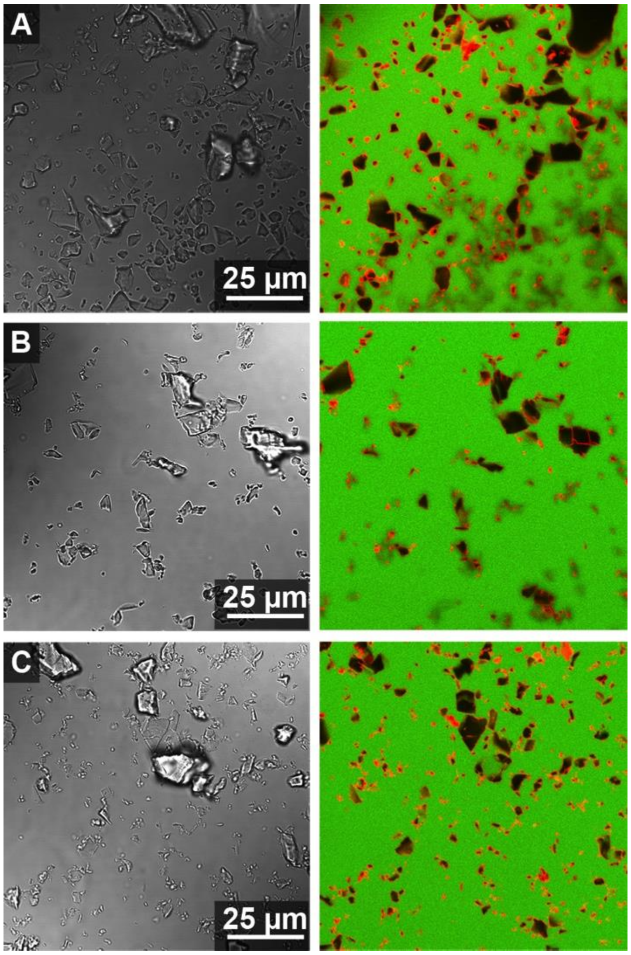

Figure S7. Interaction of materials that are probable impurities in the natural clay samples with dye-labeled PEG (green, ~80 μM) and dye-labeled Dx (red, 20 μM). Images show transmitted light (DIC, left panels) and confocal fluorescence overlay (right) for samples that contain a mixture of Alexa488-labeled PEG (green) and Alexa647-labeled Dx (red) in 100 mM HEPES buffer in the presence of (**A**) quartz, (**B**) labradorite (feldspar), and (**C**) orthoclase. Higher labeled- PEG concentration was used to observe/image any possible interaction with labeled-

PEG without any interference with labeled Dx, similar scenario with different volume ratios of ATPS.

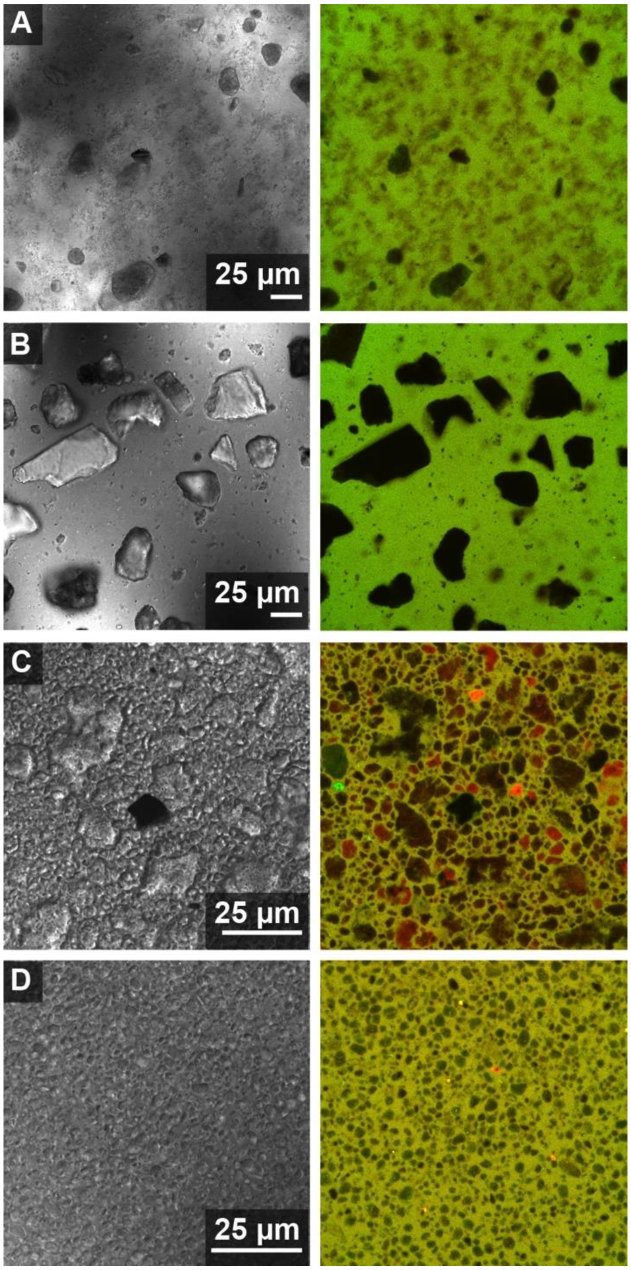

Figure S8. Interaction of natural clay particles with dyes not bound to any polymer: Alexa 488 (green) and Alexa 647 (red) with 20 μM final concentration in the presence of (**A**) Kaolinite (KGa-2), (**B**) Na-rich montmorillonite (SWy-2), (**C**) Ca-rich montmorillonite (STx-1b) and (**D**) NX-illite in 100 mM HEPES buffer pH 7.5. Left hand panels show transmitted light (DIC) and

right hand panels show overlay of confocal fluorescence from green and red channels. Higher dye concentration than general samples with labeled polymers was used to observe/eliminate any interaction with dyes.

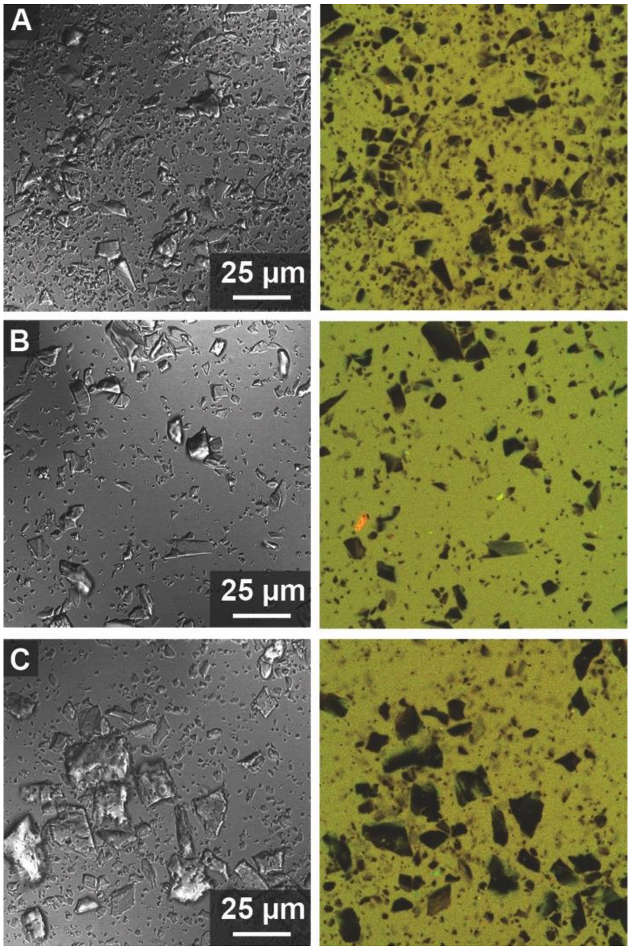

Figure S9. Dyes not bound to polymers do not accumulate at the surface of materials that are probable impurities in the natural clay samples. Images show transmitted light (DIC) and confocal fluorescence overlay for samples that contain a mixture of Alexa 488 (green) and Alexa 647 (red) dyes in 100 mM HEPES buffer with 20 μM final concentration in the presence of (**A**) quartz, (**B**) labradorite (feldspar) and (**C**) orthoclase.

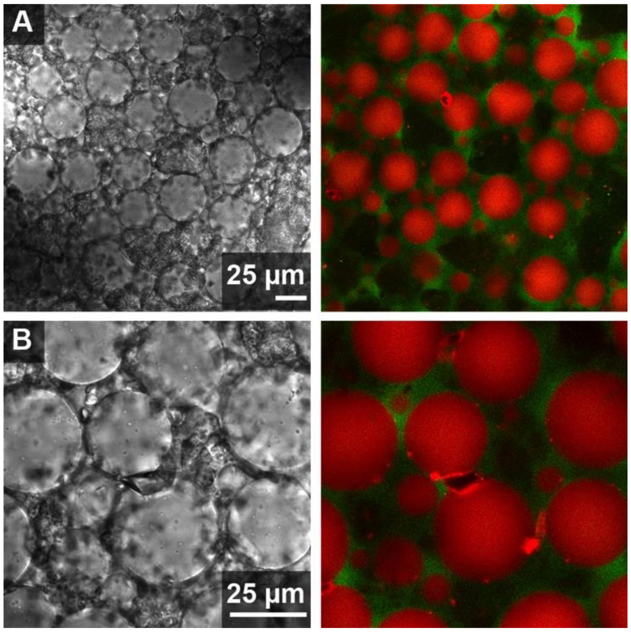

Figure S10. Montmorillonite particles accumulated between Dx-rich droplets can sterically prevent coalescence when sufficient amounts of particles are present. For 15%/15% w/w PEG 8 kDa-Dx 10 kDa using volume ratio of 20: 1 PEG-rich phase: Dx-rich phase with Ca-rich montmorillonite (STx-1b) (**A**) and (**B**). Labeled Dx (red, 2 μM final concentration) and labeled PEG (green, 2 μM final concentration) were used for fluorescence imaging. Continuous phase is PEG-rich phase while droplets are Dx-rich phase.

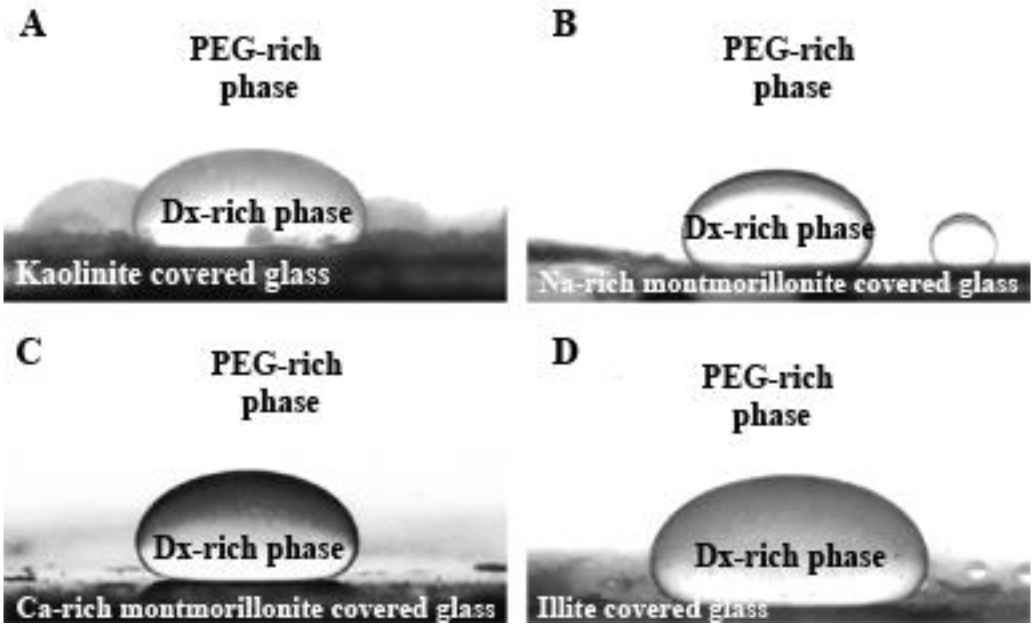

Figure S11. Representative photographs of Dx-rich phase droplets sitting on clay-covered glass slides surrounded by a PEG-rich continuous phase, collected to estimate contact angles for clay microparticles with the aqueous phases. Panels show Dx-rich droplet (3 uL) in PEG-rich continuous phase on clay-coated glass substrates for each clay: kaolinite (**A**), Na-rich montmorillonite (**B**), Ca-rich montmorillonite (**C**) and illite (**D**). Three-phase contact angles were calculated from multiple images for different Dx-rich droplets on surfaces coated with each type of clay.

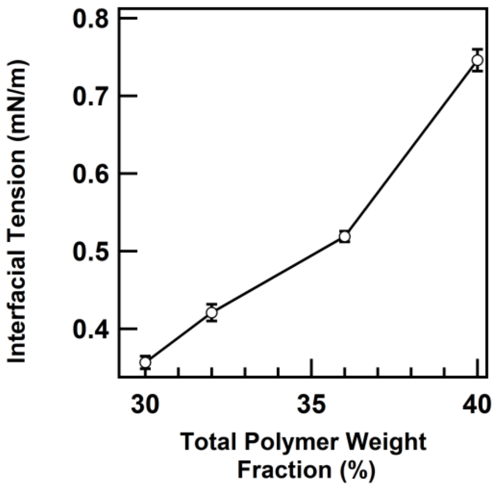

Figure S12. Surface Tension vs total polymer concentration

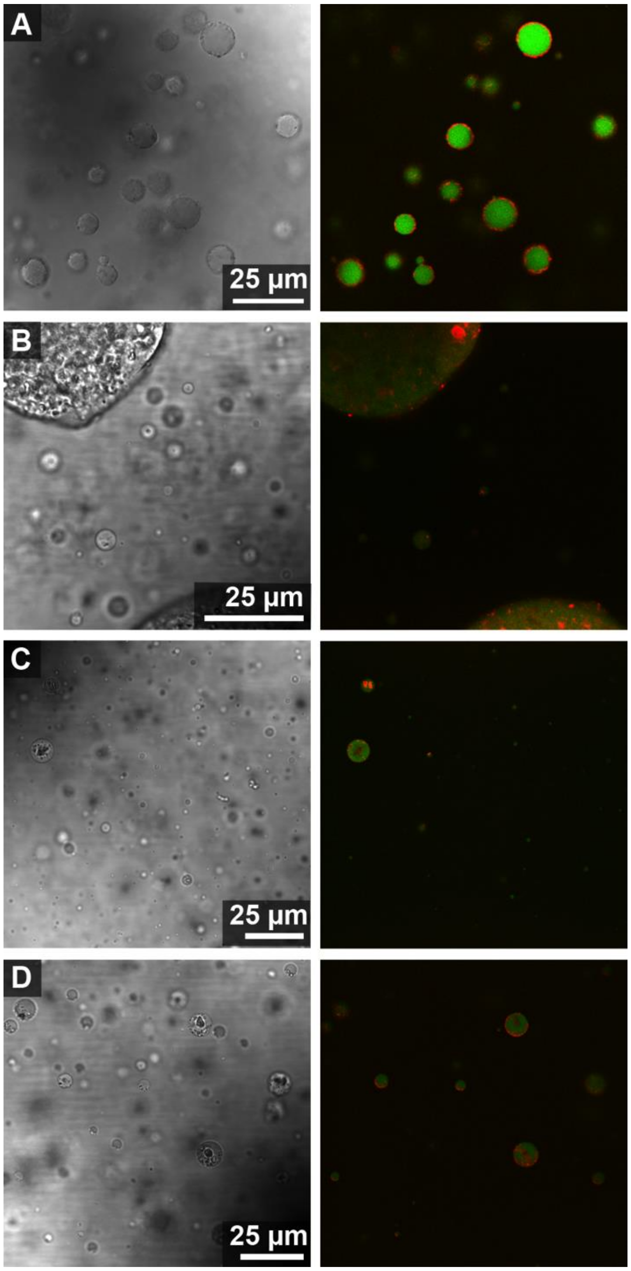

Figure S13. Clay particle distribution in ATPS with reversed volume ratio. For 15%/15% w/w PEG 8 kDa-Dx 10 kDa using volume ratio of 1: 20 PEG-rich phase: Dx-rich phase with (**A**) Kaolinite (KGa-2), (**B**) Na-rich montmorillonite (SWy-2), (**C**) Ca-rich montmorillonite (STx-1b) and (**D**) NX-illite. Labeled Dx (red, 2 μM final concentration) and labeled PEG (green, 2 μM final concentration) were used for fluorescence imaging. Continuous phase is Dx-rich phase while droplets are PEG-rich phase.

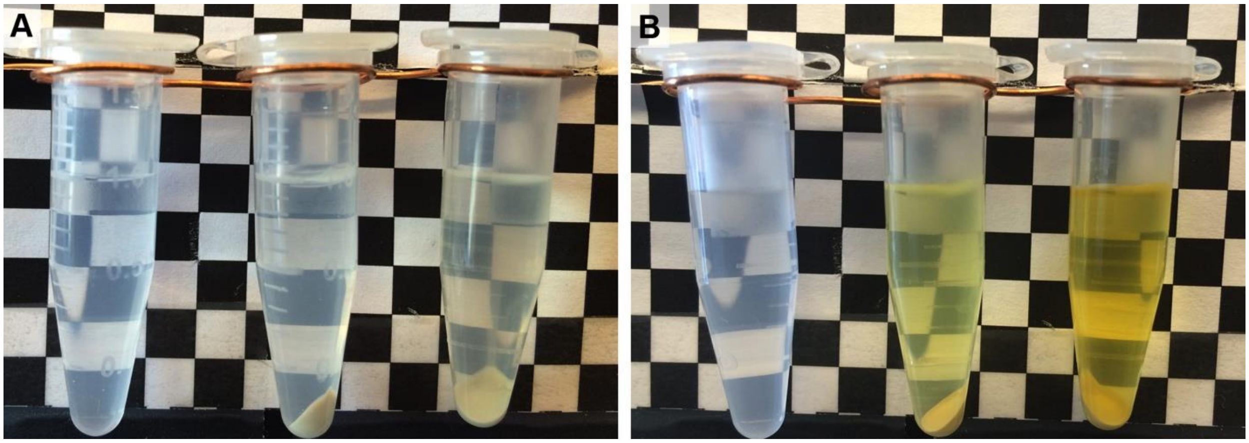

Figure S14. Reaction pictures were taken after 2 minutes (A) and 24 hours (B). Vials contain substrates in buffer without clay, substrates and illite in buffer and substrates and illite in 20:1 ratio of Dx-rich phase/PEG-rich phase, in the order from left to right respectively. Hydrogen peroxide was held in excess at 8.8 mM and OPD was 500 µM in 100 mM HEPES buffer, pH 7.5.

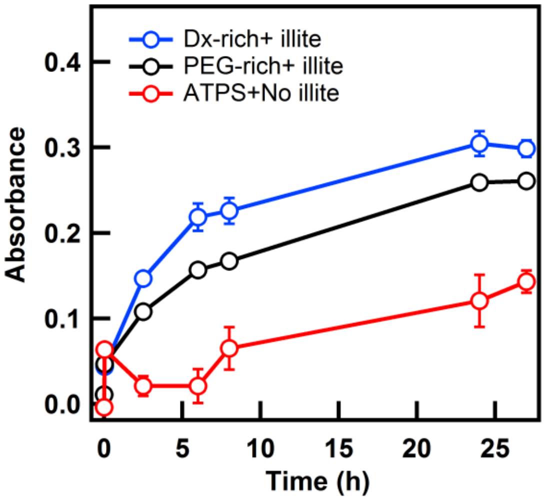

Figure S15. Reaction product absorbance at 417 nm in Dx-rich phase with illite and substrates (blue), in PEG-rich phase with illite and substrates (black) and in 20:1 ratio of Dx-rich phase/PEG-rich phase with substrates and w/o illite (red). Hydrogen peroxide was held in excess at 8.8 mM and OPD was 500 µM in 100 mM HEPES buffer, pH 7.5. Error bars represent the standard deviation of measurements.

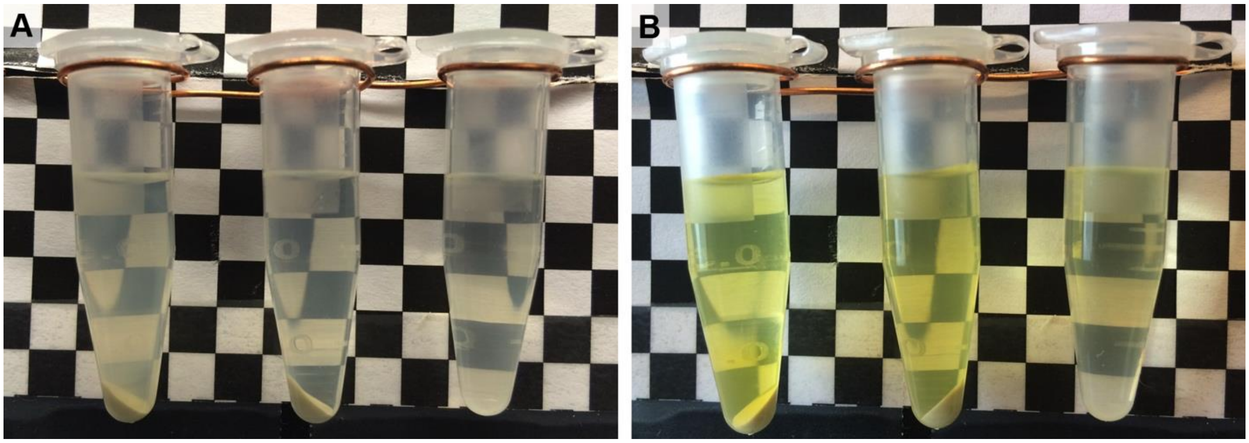

Figure S16. Reaction pictures were taken after 2 minutes (A) and 24 hours (B). Vials contain substrates and illite in Dx-rich phase, substrates and illite in PEG-rich phase and substrates but no illite in 20:1 ratio of Dx-rich phase/PEG-rich phase, in the order from left to right respectively. Hydrogen peroxide was held in excess at 8.8 mM and OPD was 500 μM in 100 mM HEPES buffer, pH 7.5.

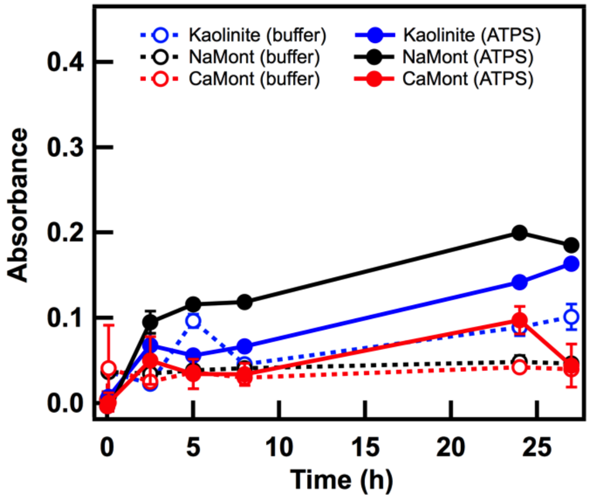

Figure S17. Reaction product absorbance at 417 nm in 20:1 ratio of Dx-rich phase/PEG-rich phase or buffer with one type of clay (kaolinite, Ca-rich, Na-rich montmorillonite) and substrates. Absorbance in PEG/Dx ATPS was graphed as solid lines in colors of blue, black and red for kaolinite, Na-rich montmorillonite and Ca-rich montmorillonite, respectively. Absorbance in buffer was graphed as dotted lines in colors of blue, black and red for kaolinite, Na-rich montmorillonite and Ca-rich montmorillonite, respectively. Hydrogen peroxide was held

in excess at 8.8 mM and OPD was 500 μM in 100 mM HEPES buffer, pH 7.5. Error bars represent the standard deviation of measurements.

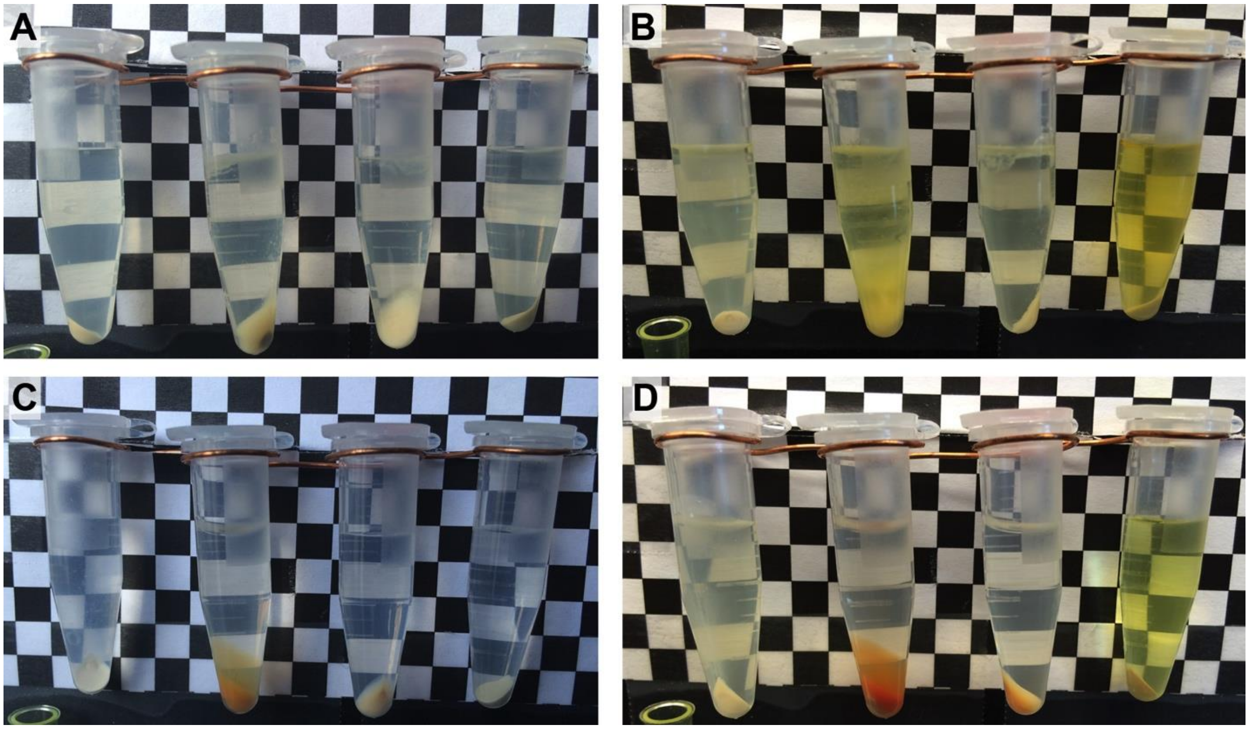

Figure S18. Reaction pictures were taken after 5 minutes (A, C) and 24 hours (B, D). Vials contain substrates and kaolinite, Na-rich montmorillonite, Ca-rich montmorillonite and illite in 20:1 ratio of Dx-rich phase/PEG-rich phase (A-B) and in buffer (C-D), in the order from left to right respectively. Hydrogen peroxide was held in excess at 8.8 mM and OPD was 500 μM in 100 mM HEPES buffer, pH 7.5.

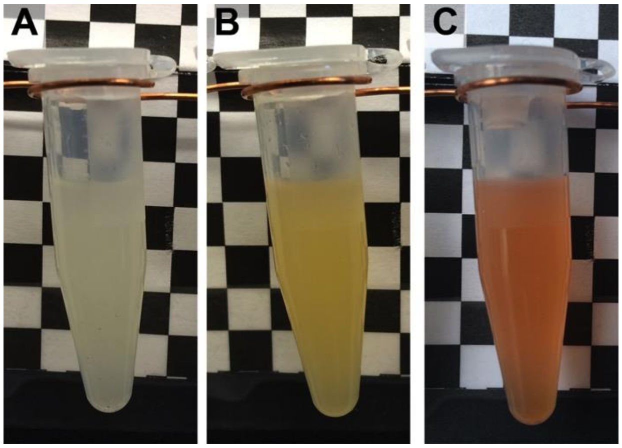

Figure S19. Reaction pictures were taken before and after addition of substrates to Na-rich montmorillonite in buffer. Pictures were taken just before (A), 5 minutes (B) and 2 hours after (C). Hydrogen peroxide was held in excess at 8.8 mM and OPD was 500 µM in 100 mM HEPES buffer, pH 7.5.

Movie Description:

Movie S 1: Z-stacking video showing red fluorescence channel for illite particles in the presence of AF 647-labeled Dx. This video shows that the dark spots seen in Figure 2D (left) are because no dye is in the center of the illite particles, rather the dye is adsorbed on the particle surface. By scanning the z-direction the dye on the particle surface (top, bottom, and sides) can be seen.

Supporting References:

1. Broadley, S. L.; Murray, B. J.; Herbert, R. J.; Atkinson, J. D.; Dobbie, S.; Malkin, T. L.; Condliffe, E.; Neve, L., Immersion mode heterogeneous ice nucleation by an illite rich powder representative of atmospheric mineral dust. *Atmos Chem Phys* **2012,** *12* (1), 287-307.

2. Strulson, C. A.; Molden, R. C.; Keating, C. D.; Bevilacqua, P. C., RNA catalysis through compartmentalization. *Nat Chem* **2012,** *4* (11), 941-946.

3. Davis, B. W.; Aumiller, W. M.; Hashemian, N.; An, S. O.; Armaou, A.; Keating, C. D., Colocalization and Sequential Enzyme Activity in Aqueous Biphasic Systems: Experiments and Modeling. *Biophys J* **2015,** *109* (10), 2182-2194.

4. Veghte, D. P.; Freedman, M. A., Facile Method for Determining the Aspect Ratios of Mineral Dust Aerosol by Electron Microscopy. *Aerosol Science and Technology* **2014,** *48* (7), 715-724.

5. VanDerVoort, P.; Vansant, E. F., Silylation of the silica surface a review. *J Liq Chromatogr R T* **1996,** *19* (17-18), 2723-2752.

6. Vis, M.; Opdam, J.; van 't Oor, I. S. J.; Soligno, G.; van Roij, R.; Tromp, R. H.; Erne, B. H., Water-in-Water Emulsions Stabilized by Nanoplates. *Acs Macro Lett* **2015,** *4* (9), 965-968.

7. Meijer, J. M.; Hagemans, F.; Rossi, L.; Byelov, D. V.; Castillo, S. I. R.; Snigirev, A.; Snigireva, I.; Philipse, A. P.; Petukhov, A. V., Self-Assembly of Colloidal Cubes via Vertical Deposition. *Langmuir* **2012,** *28* (20), 7631-7638.

8. Schneider, C. A.; Rasband, W. S.; Eliceiri, K. W., NIH Image to ImageJ: 25 years of image analysis. *Nat Methods* **2012,** *9* (7), 671-675.

9. Aumiller, W. M.; Davis, B. W.; Hatzakis, E.; Keating, C. D., Interactions of Macromolecular Crowding Agents and Cosolutes with, Small-Molecule Substrates: Effect on Horseradish Peroxidase Activity with Two Different Substrates. *J Phys Chem B* **2014,** *118* (36), 10624-10632.
